# Urinary Dopamine Levels Can Predict the Avidity of Post-Therapy [^131^I]MIBG Scintigraphy in Unresectable or Metastatic Pheochromocytomas and Paragangliomas: A Preliminary Clinical Study

**DOI:** 10.3390/ph18020165

**Published:** 2025-01-26

**Authors:** Junki Takenaka, Shiro Watanabe, Takashige Abe, Satoshi Takeuchi, Kenji Hirata, Rina Kimura, Hiroshi Ishii, Naoto Wakabayashi, Mungunkhuyag Majigsuren, Kohsuke Kudo

**Affiliations:** 1 Department of Diagnostic Imaging, Graduate School of Medicine, Hokkaido University, Sapporo 060-8638, Japankhirata@med.hokudai.ac.jp (K.H.); ms.mungunkhuyag@gmail.com (M.M.); kkudo@med.hokudai.ac.jp (K.K.); 2 Department of Nuclear Medicine, Hokkaido University Hospital, Sapporo 060-8648, Japan; 3 Global Center for Biomedical Science and Engineering, Faculty of Medicine, Hokkaido University, Sapporo 060-8638, Japan; 4 Department of Renal and Genitourinary Surgery, Graduate School of Medicine, Hokkaido University, Sapporo 060-8638, Japan; 5 Department of Medical Oncology, Faculty of Medicine, Graduate School of Medicine, Hokkaido University, Sapporo 060-8638, Japan; 6 Center of Neuroendocrine Neoplasms, Hokkaido University Hospital, Sapporo 060-8648, Japan; 7 Medical AI Research and Development Center, Hokkaido University Hospital, Sapporo 060-8648, Japan; 8 Department of Diagnostic and Interventional Radiology, Hokkaido University Hospital, Sapporo 060-8648, Japan; 9 Department of Diagnostic Radiology, National Hospital Organization, Hokkaido Cancer Center, Sapporo 003-0804, Japan; 10 Department of Radiology, Diagnostic Imaging Center, Second State Central Hospital, Ulaanbaatar 210349, Mongolia

**Keywords:** PPGL, [^131^I]MIBG therapy, urine catecholamine

## Abstract

**Background/Objectives**: Pheochromocytomas and paragangliomas (PPGLs) are rare neuroendocrine tumors that produce catecholamines. Unresectable or metastatic PPGLs are treated with [^131^I]metaiodobenzylguanidine (MIBG), but MIBG avidity is often heterogeneous. Identifying predictive factors for non-avid lesions on scintigraphy is clinically important. The primary objective of this study was to investigate the relationship between MIBG avidity and catecholamine secretion patterns in patients with unresectable or metastatic PPGLs. **Methods**: This retrospective study included 27 patients treated with [^131^I]MIBG for unresectable/metastatic PPGLs between 2001 and 2024. Patients received a single intravenous dose of [^131^I]MIBG (5.5–7.4 GBq), with post-therapy scintigraphy performed 3–7 days later. Non-avid lesions were assessed by imaging and confirmed using CT, MRI, and FDG-PET. Clinical factors, including age, sex, prior treatments, metastasis sites, and urine catecholamines, were evaluated using univariate logistic analysis. Predictive factors were assessed via receiver operating characteristic curves. **Results**: Non-avid lesions were found in nine patients (33.3%). These patients were younger (median age 38 vs. 62.5 years) and had higher urine dopamine levels (median 1510 vs. 779 μg/day) than those without non-avid lesions. Younger age (odds ratio: 0.892, *p* < 0.01) and higher urinary dopamine levels (odds ratio: 1.003, *p* < 0.01) were significantly associated with non-avid lesions. All patients > 45 years with urinary dopamine < 1190 μg/day had no non-avid lesions, whereas patients < 45 years with urinary dopamine > 1190 μg/day had non-avid lesions. **Conclusions**: Age and urinary dopamine levels may predict non-avid lesions in unresectable/metastatic PPGLs, aiding treatment decisions for [^131^I]MIBG therapy. This article is a revised and expanded version of a paper entitled “Urine dopamine level and age can predict non-avid lesion on scintigraphy after I-131 MIBG treatment for unresectable/metastatic PPGL”, which was presented at SNMMI 2024, Toronto, from 8 June to 11 June 2024.

## 1. Introduction

Pheochromocytomas and paragangliomas (PPGLs) are rare neuroendocrine tumors that produce varying levels of catecholamines. The 2022 World Health Organization classification defines pheochromocytomas as tumors arising from the adrenal medulla, and paragangliomas as tumors arising from extra-adrenal chromaffin tissues [1]. The annual incidence of PPGLs is estimated to range from 0.04 to 0.66 cases per 100,000 individuals [2,3]. All PPGLs have malignant potential, with approximately 15–20% eventually developing metastases [4]. The 5-year survival rates for PPGLs are reported to range from 75.4% to 91% [5,6].

Catecholamine secretion patterns reflect tumor differentiation and prognosis. Patients with elevated dopamine and noradrenaline levels tend to exhibit higher metastatic rates and poorer outcomes [7]. Advances in molecular classification have further stratified PPGLs into three genetic clusters. Cluster 1 includes mutations stabilizing hypoxia-inducible factor-1 (HIF-1), cluster 2 involves mutations in tyrosine kinase pathways, and cluster 3 includes mutations in the Wnt signaling pathway [8]. Among these, cluster 1A tumors are the most aggressive, with the highest metastatic rates, requiring timely intervention in 10–15% of cases [9,10,11,12]. Cluster 1 tumors are also associated with the noradrenaline and dopaminergic secretion pattern [8].

Surgical resection remains the definitive treatment for PPGLs. However, when lesions become unresectable, recurrent, or metastatic, alternative therapeutic strategies must be considered [13,14]. Observation may be an option for select cases, while locoregional treatments such as surgery, radiation therapy, or radiofrequency ablation can be employed when applicable. Systemic treatment options include [^131^I]metaiodobenzylguanidine ([^131^I]MIBG) therapy, peptide receptor radionuclide therapy (PRRT), and chemotherapy, such as the cyclophosphamide, vincristine, and dacarbazine (CVD) regimen [13,14].

Functional imaging plays a pivotal role in the precision medicine approach to PPGLs, aiding in their localization and specific characterization due to the unique characteristics of PPGL cell membranes [15,16]. Scintigraphy utilizes single-photon-emitting radiopharmaceuticals such as [^123^I]/[^131^I]MIBG for nuclear imaging. In contrast, PET imaging relies on detecting two coincident high-energy photons emitted by positron-emitting radioisotopes, such as [^18^F]FDG, [^18^F]DOPA, and [^68^Ga]/[^64^Cu] DOTA-TATE/TOC/NOC [15,16]. Among these, [^68^Ga]/[^64^Cu] DOTA-TATE/TOC/NOC demonstrates the highest sensitivity and is critical for determining the suitability of peptide receptor radionuclide therapy (PRRT) [17,18]. [^18^F]FDG is useful for metastatic surveys, particularly in tumors with disrupted Krebs cycles due to SDHx mutations [19,20], while [^18^F]DOPA is effective in detecting metastases in VHL-, EPAS1 (HIF2A)-, and FH-associated PPGLs [21,22]. Although MIBG scintigraphy is less sensitive than other modalities, it remains crucial for determining the indication for [^131^I]MIBG therapy [15,23].

MIBG, an analog of noradrenaline (norepinephrine), is selectively concentrated in tumors originating from neuroectodermal tissues, including PPGLs, through noradrenaline transporters [24]. [^131^I]MIBG treatment is effective for unresectable or metastatic PPGLs, and its adverse effects are relatively mild [25,26,27]. Since response rates to [^131^I]MIBG treatment have been reported to be approximately 30% [28], an appropriate selection of cases for treatment is necessary.

The dopaminergic pattern has been linked to poor treatment response and prognosis following [^131^I]MIBG therapy [29,30], as tumors with this pattern are thought to exhibit low MIBG accumulation. However, direct measurement of the absorbed dose of [^131^I]MIBG remains challenging due to difficulties in acquiring planar imaging or single-photon emission computed tomography (SPECT). In this study, we evaluated whether patients exhibited non-avid lesions on scintigraphy following [^131^I]MIBG therapy. Our objective was to investigate the relationship between MIBG avidity and catecholamine secretion patterns in patients with unresectable or metastatic PPGLs.

## 2. Results

### 2.1. Patient Characteristics

**Figure 1:**
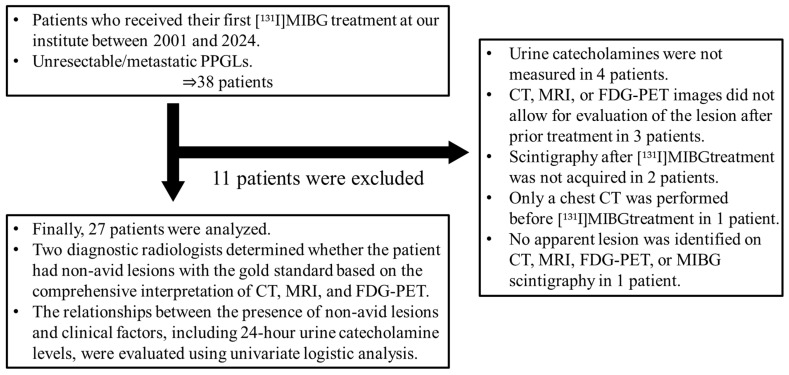
Flow diagram of the participant inclusion process.

The final population for subsequent analyses consisted of 27 patients. We initially reviewed 38 patients with unresectable or metastatic PPGL who underwent [^131^I] MIBG therapy at our institution between 2001 and 2024. Of these, we excluded four patients due to unavailability of urine catecholamine data; three patients due to missing computed tomography (CT), magnetic resonance imaging (MRI), or fluorodeoxyglucose (FDG)-positron emission tomography (PET) images required for lesion evaluation after prior treatment; two patients due to the lack of post-therapy [^131^I]MIBG scintigraphy; one patient who only underwent chest CT before [^131^I]MIBG treatment; and one patient with no identifiable lesion on CT, MRI, FDG-PET, or MIBG scintigraphy (Figure 1).

**Figure 2:**
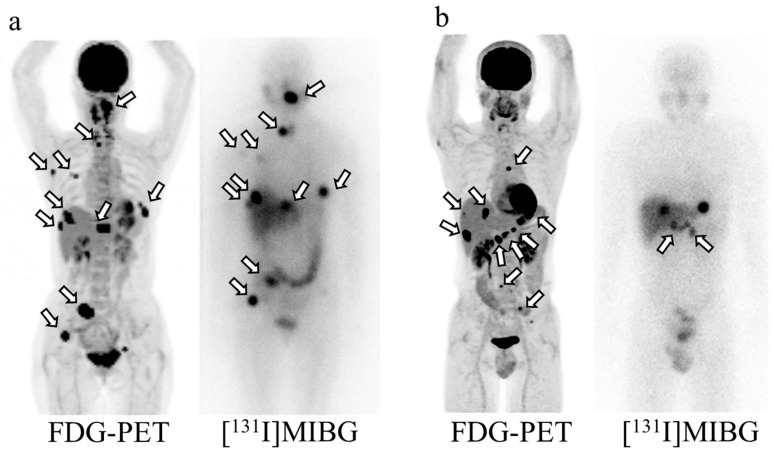
Representative cases. (**a**) The MIP image of the FDG-PET prior to [^131^I]MIBG treatment of a 65-year-old woman with bone and lymph node metastases. The urine catecholamines included adrenaline (14.3 μg/day), noradrenaline (1110.2 μg/day), and dopamine (989.2 μg/day). All of the metastases showed apparent uptake on scintigraphy after [^131^I]MIBG treatment. (**b**) The MIP image of the FDG-PET prior to [^131^I]MIBG treatment of a 45-year-old man with lymph node, liver, and bone metastases. The urine catecholamines included adrenaline (7.9 μg/day), noradrenaline (647.5 μg/day), and dopamine (2115.9 μg/day). Only a few lymph node metastases showed apparent uptake on scintigraphy after [^131^I]MIBG treatment_._ MIP, maximum intensity projection; FDG-PET, fluorodeoxyglucose-positron emission tomography; MIBG, metaiodobenzylguanidine.

Patient characteristics are summarized in Table 1. Among the 27 patients included in the study, 9 patients (33.3%) had non-avid lesions. Of these, four patients (14.8%) exhibited no avid lesions, while the remaining five patients had both avid and non-avid lesions. Their ages ranged from 23–84 years. Eleven (40.7%) of the twenty-seven patients were male. Seventeen (63.0%) patients had pheochromocytomas, and the other ten (37.0%) had paragangliomas. Primary tumors, peritoneal dissemination, and lymph node metastases were resected as extensively as possible prior to [^131^I]MIBG treatment. All 27 patients had imaging evidence of the tumor, including a residual primary tumor (*n* = 2) or metastasis to the lymph nodes or soft tissue (*n* = 15), bone (*n* = 16), liver (*n* = 12), or lungs (*n* = 11). Non-avid lesions included residual primary tumors (*n* = 1) and metastatic lesions in the lymph nodes or soft tissue (*n* = 5), bone (*n* = 4), liver (*n* = 2), or lungs (*n* = 2). Eight (29.6%) patients received treatment with cyclophosphamide, vincristine, and dacarbazine prior to [^131^I]MIBG treatment; one (7.4%) patient received [^131^I]MIBG therapy at another hospital at a dose of 7.4 GBq prior to [^131^I]MIBG treatment at our institution; and eight patients (29.6%) received external radiation for bone metastases. Two representative cases are presented in Figure 2.

### 2.2. Biochemical Parameters

**Figure 3:**
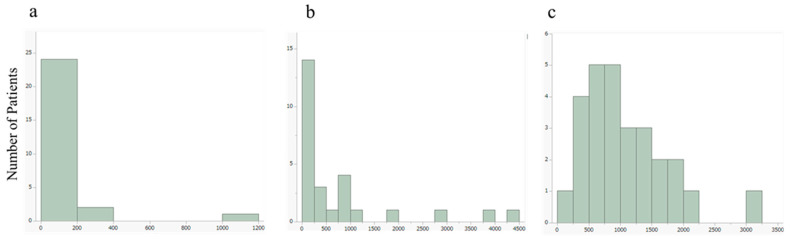
Bar charts illustrating urinary catecholamine levels: (**a**) adrenaline, (**b**) noradrenaline, and (**c**) dopamine.

The 24 h urine collection was conducted a median of 2 days before [^131^I]MIBG treatment (range: 0–58 days). As shown in Table 2, the median urinary adrenaline level was 6.9 μg/day (range: 1.8–1080 μg/day; interquartile range [IQR]: 3.3–13.3 μg/day; mean ± SD: 63.4 ± 211.6 μg/day), with a normal reference range of 3.4–26.9 μg/day. The median urinary noradrenaline level was 224.3 μg/day (range: 59.1–4282 μg/day; IQR: 171.6–930.0 μg/day; mean ± SD: 790.3 ± 1138.8 μg/day), compared to the normal range of 48.6–168.4 μg/day. The median urinary dopamine level was 963.2 μg/day (range: 249.9–3187.5 μg/day; IQR: 618.4–1492.4 μg/day; mean ± SD: 1103.8 ± 658.0 μg/day), with a normal range of 365.0–961.5 μg/day. Figure 3 presents bar charts illustrating urinary catecholamine levels, including adrenaline, noradrenaline, and dopamine (Figure 3).

### 2.3. Assessment of MIBG Avidity and Clinical Information Including Urinary Catecholamine Levels

**Figure 4:**
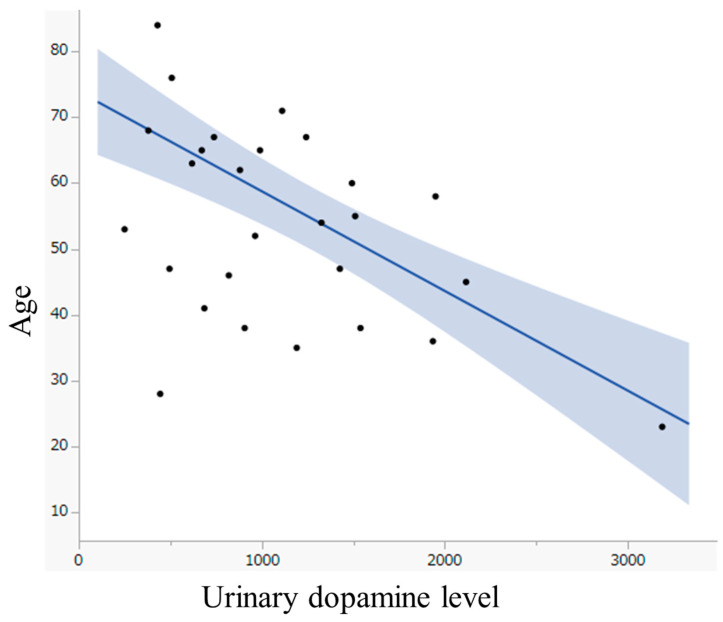
Scatter plot depicting the relationship between age and urinary dopamine levels with a regression line.

The results of the univariate logistic analysis evaluating MIBG avidity and clinical information, including urinary catecholamine levels, are presented in Table 3. Patients with non-avid lesions were younger than those without non-avid lesions, with a median age of 38 years (range: 23–60 years) and 62.5 years (range: 38–84) years, respectively. The median urine dopamine level of the patients with non-avid lesions was 1510 μg/day (range: 445–3188 μg/day), which was higher than 779 μg/day (range: 250–1949 μg/day) for patients without non-avid lesions. Younger age (odds ratio: 0.892, *p* < 0.01) and higher urinary dopamine levels (odds ratio: 1.003, *p* < 0.01) were significant predictive factors for the presence of non-avid lesions on scintigraphy after [^131^I]MIBG treatment (Table 3). These two significant predictive factors were not statistically correlated (ρ = −0.373, *p* = 0.056, Figure 4), and the remaining clinical factors were not associated with [^131^I]MIBG avidity.

**Figure 5:**
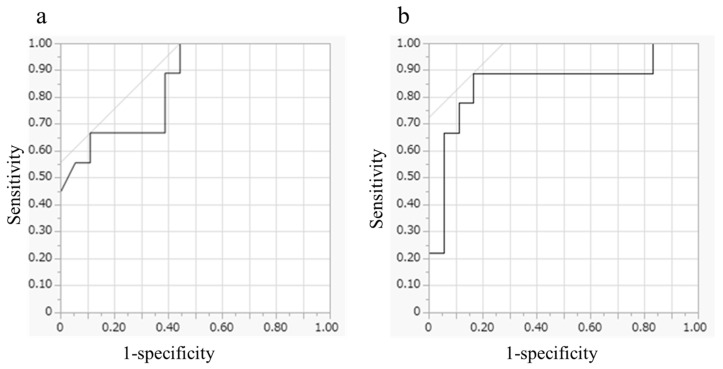
ROC curves for (**a**) age and (**b**) urinary dopamine level. ROC, receiver operating characteristic.

The optimal cut-off values for predicting non-avid lesions on scintigraphy following [^131^I]MIBG treatment were determined using ROC-area under the curve (AUC) (Figure 5). The cutoff values for age were identified as either 45 or 60 years (AUC: 0.849; sensitivity of 66.7% and specificity of 88.9% and sensitivity of 100% and specificity of 55.6%, respectively). For urinary dopamine, the cut-off value was 1190 μg/day (AUC: 0.852; sensitivity of 88.9% and specificity of 83.3%). All patients aged >45 years with urinary dopamine levels < 1190 μg/day did not have non-avid lesions (*n* = 13), whereas patients <45 years with urinary dopamine levels > 1190 μg/day had non-avid lesions (*n* = 5) (Table 4). Even when analyzed using Fisher’s exact test, no significant relationship was observed between the two variables (*p* = 0.21).

## 3. Discussion

We investigated the clinical factors predicting whether patients had non-avid lesions on post-therapy [^131^I]MIBG scintigraphy in this retrospective analysis of 27 patients with PPGLs. Our findings indicated that it may be possible to estimate the presence of non-avid lesions after [^131^I]MIBG scintigraphy by using the pre-treatment urine dopamine level and age of the patients.

The results of this study are considered highly relevant to clinical practice. While patients undergo [^123^I]MIBG scintigraphy prior to [^131^I]MIBG treatment to determine eligibility, previous studies have shown that post-treatment scintigraphy using [^131^I]MIBG provides superior diagnostic performance compared to pre-treatment evaluation with [^123^I]MIBG [31]. This underscores the practical importance of post-treatment evaluation in optimizing clinical outcomes.

PPGL are rare neuroendocrine tumors that produce catecholamines. Catecholamine biosynthesis proceeds through a series of enzymatic reactions that convert tyrosine into dihydroxyphenylalanine and then into dopamine, noradrenaline, and adrenaline [32]. PPGL can be classified into adrenergic, noradrenergic, and dopaminergic phenotypes, wherein noradrenergic and dopaminergic phenotypes indicate enzyme defects and are regarded as immature compared with the adrenergic type [7,32,33]. It has been suggested that patients with elevated dopamine and noradrenaline secretion not only have a higher frequency of metastasis [7,34] but also have a poorer prognosis compared to those without elevated dopamine and noradrenaline secretion [29]. [^131^I]MIBG treatment is effective and safe for unresectable or metastatic PPGLs and is recommended by the ESMO as a first-line treatment [14]; however, the treatment response to [^131^I]MIBG has been shown to be significantly poor in patients with elevated urinary dopamine levels [30], likely due to poor accumulation of [^131^I]MIBG, which is congruent with the results of the current study.

The dopaminergic phenotype likely corresponds to a pseudohypoxia cluster, which is characterized by the activation of pathways that mimic hypoxic signaling, including mutations in succinate dehydrogenase, fumarate hydratase, and isocitrate dehydrogenase [8,35]. This cluster of PPGLs tend to occur at younger ages and is frequently associated with poor MIBG avidity [8,36], which is consistent with our findings. MIBG, a noradrenaline analog, accumulates in PPGLs cells via the uptake-1 noradrenaline transporter [24]. Dopamine-producing PPGLs are thought to exhibit reduced uptake-1 expression; however, the mechanism by which specific gene mutations lead to reduced uptake-1 expression remains unclear.

A previous study reported that urinary 3-methoxytyramine excretion was increased in 31 (23%) of the 136 patients with head and neck paraganglioma who were examined. Among these, only 11 (41%) of the 27 patients who underwent MIBG scintigraphy demonstrated MIBG accumulation [37]. This finding is consistent with our research and highlights a potential link between catecholamine metabolites and MIBG uptake.

A related study [38] demonstrated that homovanillic acid (HVA), a final metabolite of dopamine, is associated with decreased cardiac accumulation of MIBG in neuroblastoma patients. This observation suggests that HVA, or the dopamine metabolic pathway, may competitively inhibit MIBG cardiac uptake via the noradrenaline transporter. A similar mechanism may account for the reduced MIBG uptake observed in patients with unresectable or metastatic pheochromocytoma and paraganglioma (PPGL) who exhibit elevated dopamine levels. However, since MIBG is an analog of noradrenaline, tumors producing noradrenaline may exhibit variable uptake. Despite this, such tumors have been reported to frequently demonstrate MIBG accumulation [39].

The results of the present investigation suggest that other effective treatments should be applied to high dopamine-producing PPGLs. Several retrospective studies have suggested that this type of PPGLs show strong somatostatin receptor expression and that peptide receptor radionuclide therapy (PRRT) is one of the most effective clinical therapies for PPGL [8,40,41]. Meta-analyses have shown that the response rate of PRRT is approximately 25% [42]; thus, PRRT is considered an effective treatment for PPGLs.

### Study Limitations

This was a single-center retrospective analysis, and the number of participants (*n* = 27) was limited. Due to the small sample size, it was not possible to perform a multivariate analysis. We did not evaluate the catecholamine metabolites of patients, including metanephrines, normetanephrines, and 3-methoxytyramine (3-MT), which are considered more reliable markers than catecholamines themselves [8,35,43,44]; 3-MT is generally unavailable in Japan, while metanephrines and normetanephrines were not analyzed in this study because of missing data. A lesion-by-lesion assessment of [^131^I]MIBG scintigraphy uptake was not performed due to the low image quality of [^131^I]MIBG scintigraphy. This assessment can be conducted using [^124^I]MIBG [45] or [^18^F]MFBG [46]; however, these modalities are not widely available in Japan. While these PET scans demonstrate excellent diagnostic capability, the uptake of [^131^I]MIBG may differ from that observed in PET imaging. The collection time window for the patient’s urine samples was relatively broad, reflecting the retrospective nature of a study conducted under routine clinical practice. However, such an extended period raises concerns, as a sample collected 58 days after the initial event may obscure significant changes, including substantial tumor progression. Genetic mutations in SDHB were not evaluated in the present study because examining gene mutations presents a dilemma, as they occur in germ cells. The results can have profound implications not only for the patients themselves but also for their blood relatives. Although the sample size of our study is small and lesion assessments may have limitations, this research is valuable for understanding this rare disease. Future studies should address these limitations to further validate our findings.

## 4. Materials and Methods

This retrospective study was approved by the Institutional Review Board of Hokkaido University Hospital (#022-0329) based on the principles of the World Medical Association’s Declaration of Helsinki. The requirement for written informed consent was waived due to the retrospective study design.

### 4.1. Patients

The inclusion criteria consisted of patients with unresectable or metastatic PPGL who underwent [^131^I]MIBG therapy at our institution between 2001 and 2024. The exclusion criteria included the following: unavailability of urine catecholamine data; lack of appropriate imaging evaluation CT, MRI, or FDG-PET that allowed for lesion assessment after prior treatment; unavailability of post-therapy [^131^I]MIBG scintigraphy; or absence of apparent lesions identifiable on CT, MRI, FDG-PET, or MIBG scintigraphy.

### 4.2. [^131^I]MIBG Treatment

The [^131^I]MIBG was provided by Izotop (Institute of Isotopes Co., Budapest, Hungary; *n* = 3; August 2008 to March 2009), POLATOM (National Center for Nuclear Research Radioisotope Center, Otwock, Poland; *n* = 20, April 2009 to November 2020), and PDR Pharma (Institute of Isotopes Co., Tokyo, Japan; *n* = 4; December 2020 to March 2024). A single intravenous dose of [^131^I]MIBG (5.5–7.4 GBq) was administered over a 1 h period. Patients receiving [^131^I]MIBG were isolated in a room dedicated to radionuclide therapy until they met the legal criteria in Japan.

Before and after [^131^I]MIBG administration, potassium iodide was orally administered to prevent radiation exposure to the thyroid. Potassium iodide (300 mg) was administered orally for 7 days, starting one day before treatment. [^131^I]MIBG scintigraphy was performed within 2 days from the end of the isolation period.

### 4.3. Biochemical Parameters (Urine Biochemistry)

The 24 h urinary catecholamine levels, including adrenaline, noradrenaline, and dopamine, were measured using urine samples collected over a 24 h period prior to the initiation of treatment. Hydrochloric acid was used as a preservative to prevent catecholamine degradation. The urine samples were collected a median of 2 days before [^131^I]MIBG therapy (range: 0–58 days).

### 4.4. [^131^I]MIBG Scintigraphy Acquisition and Analysis

Whole-body images were acquired using a dual-head γ-camera equipped with high-energy general-purpose collimators for [^131^I]MIBG. The imaging systems used included the Millennium (GE Healthcare, Chicago, IL, USA; 2008–2016), Symbia Evo (Siemens Healthineers, Erlangen, Germany; 2016–2024), and Symbia Intevo (Siemens Healthineers, Erlangen, Germany; 2020–2024). In the Millennium system, overlapping anterior and posterior images were acquired for either 100,000 counts or for 20 min, whichever threshold was reached first. In contrast, with the Symbia Evo and Symbia Intevo systems, overlapping anterior and posterior images were acquired over a fixed duration of 15 min.

Scintigraphy after [^131^I]MIBG was performed after a median of 4 days (range: 3–7 days). Whole-body images were evaluated independently by two diagnostic radiologists (one with 22 years of experience and the other with 7 years of experience) to determine whether the patient had non-avid lesions, with the ground truth based on the comprehensive interpretation of the CT, MRI, and FDG-PET. Lesions were classified as non-avid if nuclear medicine physicians assessed their uptake as less than the background activity. When the evaluations of the two radiologists differed, a third diagnostic radiologist with 13 years of experience participated, and discrepancies were resolved through discussion.

### 4.5. Statistical Analyses

Statistical calculations were performed using JMP^®^ ver. 17.0 (SAS, Cary, NC, USA). Clinical factors, including age at initial treatment, sex, disease type (PPGLs), history of prior chemotherapy and external radiotherapy, number of organs with metastatic lesions, and 24 h urine catecholamine levels, were evaluated using univariate logistic analysis to predict the presence of non-avid lesions. The relationships between significant predictive factors for MIBG avidity were evaluated using Spearman’s rank correlation coefficients and Fisher’s exact test. Furthermore, we evaluated the diagnostic performance of significant predictive factors using receiver operating characteristic (ROC) curves. Statistical significance was set at *p* < 0.05.

## 5. Conclusions

[^131^I] MIBG therapy is one of the most essential treatments for unresectable or metastatic PPGLs. Our findings suggest that non-avid lesions on scintigraphy after [^131^I] MIBG treatment may be predicted using patient age and pre-treatment urinary dopamine levels. However, the variability in catecholamine measurements and the potential influence of other factors, such as tumor burden, must be considered. Further studies with larger sample sizes and multicenter designs are required to validate these results and explore the underlying mechanisms.

## Figures and Tables

**Table 1:** Patient characteristics.

Males	11	40.7%
Females	16	59.3%
Age, median (range)	54 (23–84)	
Diagnosis		
Pheochromocytoma	17	63.0%
Paraganglioma	10	37.0%
Prior treatment		
Surgery	26	96.3%
Chemotherapy	8	29.6%
External radiation	8	29.6%
MIBG	2	7.4%
Radiofrequency ablation	2	7.4%
Endovascular treatment	1	3.7%
Residual primary tumor	2	7.4%
Metastasis		
Lymph node or soft tissue	15	55.6%
Bone	16	59.3%
Liver	12	44.4%
Lung	11	40.7%
Metastasis (no. of organs):		
1	10	37.0%
2	10	37.0%
3	5	18.5%
4	2	7.4%
Patients who had non-avid lesions on scintigraphy after [^131^I]MIBG treatment	9	33.3%

MIBG, metaiodobenzylguanidine.

**Table 2:** Urine biochemistry parameters (μg/day).

	Median	Range	IQR	Mean	SD	Normal Range
Adrenaline	6.9	1.8–1080	3.3–13.3	63.4	211.6	3.4–26.9
Noradrenaline	224.3	59.1–4282	171.6–930.0	790.3	1138.8	48.6–168.4
Dopamine	963.2	249.9–3187.5	618.4–1492.4	1103.8	658	365.0–961.5

IQR, interquartile range; SD, standard deviation.

**Table 3:** Univariate logistic analysis of MIBG avidity of patients with and without non-avid lesions on [^131^I]MIBG scintigraphy.

Variable	With Non-Avid Lesions	Without Non-Avid Lesions	OR (95% CI)	*p*-Value
Number	9	18		
Sex				
Man	5	6	0.250 (0.485–12.89)	0.273
Woman	4	12		
Age (y), median (range)	38 (23–60)	62.5 (38–84)	0.892 (0.816–0.975)	0.0014 *
Diagnosis				
Pheochromocytoma	4	13	0.308 (0.053–1.604)	0.162
Paraganglioma	5	5		
History of chemotherapy	3	5	1.300 (0.211–7.307)	0.767
History of external radiation	3	5	1.300 (0.211–7.307)	0.767
Metastatic lesion				
Lymph node or soft tissue	6	9	2.000 (0.393–11.92)	0.408
Bone	5	11	0.795 (0.154–4.198)	0.782
Liver	3	9	0.500 (0.084–2.543)	0.408
Lung	3	8	0.625 (0.118–3.316)	0.581
Urine biochemistry				
Adrenarine (μg/day), median (range) mean ± SD	6.7 (3.2–12.30) 6.63 ± 2.898	9 (2–1080) 91.75 ± 256.7	0.947 (0.787–1.139)	0.086
Noradrenaline (μg/day), median (range) mean ± SD	213.9 (116.2–961.9) 354.81 ± 286.3	249 (59–4282) 1008 ± 1339	0.999 (0.997–1.000)	0.090
Dopamine (μg/day), median (range) mean ± SD	1510 (445–3188) 1637.7 ± 748.9	779 (250–1949) 836.87 ± 414.6	1.003 (1.001–1.006)	0.0011 *

MIBG, metaiodobenzylguanidine; OR, odds ratio; CI, confidence interval; SD, standard deviation. * *p* ≤ 0.05.

**Table 4:** Distribution of dopamine levels across age groups.

	Dopamine Levels >1190 μg/Day	Dopamine Levels <1190 μg/Day	
Age < 45 years	5	3	8
Age > 45 years	6	13	19
	11	16	27

## Data Availability

Data supporting this manuscript can be accessed by contacting the corresponding author upon reasonable request.
